# Distribution of *emm* types and superantigens among group A *Streptococcus* isolates recovered from northern Tshwane, South Africa

**DOI:** 10.4102/sajid.v40i1.714

**Published:** 2025-09-20

**Authors:** Matete O. Kgasha, Skuvet T. Mashailane, Xongani V. Khosa, John Y. Bolukaoto, Marie C. le Roux, Maphoshane Nchabeleng

**Affiliations:** 1 Department of Microbiological Pathology, School of Medicine, Sefako Makgatho Health Sciences University, Pretoria, South Africa; 2 Department of Medical Microbiology, Dr George Mukhari Academic Laboratory, National Health Laboratory Service, Pretoria, South Africa

**Keywords:** *Emm* type, group A *Streptococcus*, M-proteins, superantigen, vaccine type

## Abstract

**Background:**

Group A *Streptococcus* (GAS) is a human pathogen that causes various diseases ranging from localised infections to toxin- and immune-mediated conditions.

**Objectives:**

We aimed to describe the prevalence of GAS pharyngitis in northwestern Tshwane and to characterise GAS isolates from this region.

**Method:**

GAS isolates were obtained from throat swabs of patients presenting with symptoms of pharyngitis. Clinical isolates were also collected from the DGM Laboratory. Minimum inhibitory concentrations for penicillin, erythromycin and clindamycin were determined using the E-test method. M-protein (*emm*) typing and superantigens (SAgs) profiles were determined using conventional PCR and sequencing.

**Results:**

Among the 400 throat swabs collected, 33 (8%) tested positive for GAS on culture. Additionally, 72 clinical isolates were obtained. Overall, 105 isolates were available, of which 8 (7.6%) were invasive and 97 (92.4%) were non-invasive. All the isolates were susceptible to the tested antibiotics. Twenty-seven *emm* types were identified, with *emm*82 being the most prevalent (15%). The potential vaccine coverage among the isolates was 11%. The SAg profiles identified were K and Q.

**Conclusion:**

The prevalence of GAS pharyngitis was 8%. No antimicrobial resistance detected. *Emm* typing showed significant diversity, with more than half of the isolates not covered by the 30-valent M-protein vaccine. The most prevalent was *emm*82, with speH and speI SAgs equally prevalent in these isolates. The main SAg profiles identified were K and Q. The diversity of these virulence factors suggests that it would be a challenge to consider them as potential vaccine candidates in this region.

**Contribution:**

These epidemiological findings offer significant data on the *emm* types and SAgs in GAS isolates circulating in the region, which may inform the development of an effective vaccine.

## Introduction

Group A *Streptococcus* (GAS; *Streptococcus pyogenes*) is a strict human pathogen of global health importance, responsible for a wide range of suppurative and nonsuppurative infections.^1,2,3,4^ These include superficial, locally non-invasive diseases such as pharyngitis, as well as serious and systemic infections such as bacteremia and streptococcal toxic shock syndrome.^1,2,3^ The global burden of invasive GAS infections is estimated to be at least 663 000 new cases and 163 000 deaths annually.^5,6^ There are more than 111 million cases of GAS pyoderma and more than 616 million cases of pharyngitis annually.^7,8,9^ Group A *Streptococcus* accounts for 5% – 15% of pharyngitis cases in adults and 20% – 40% in children in the United States (US).^8^ However, among the African countries, the proportions of pharyngitis cases attributed to GAS range from 9.3% in Morocco to 41.3% in Tunisia.^6^ Group A *Streptococcus* – infections may also trigger the immune-mediated disease acute rheumatic fever (ARF), and repeated episodes of ARF can lead to permanent damage to the heart valves, resulting in rheumatic heart disease (RHD).^1,2,3,4,10^ In developing countries, the prevalence of ARF and RHD is highly underreported. Several countries, including South Africa, have been making efforts to contain the burdens of ARF and RHD.^11^

Group A *Streptococcus* is a human adaptive bacterial pathogen that produces both surface-expressed and secreted virulence factors and colonises the skin and throat.^7^ These virulence factors are vital for establishing infections.^12^ The M-protein, a key virulence factor of GAS, is a fimbrial surface protein encoded by the *emm* gene.^13^ Variation of the gene is used as the basis for GAS genotyping.^13^ The N-terminal region of the M-protein contains a highly variable amino acid sequence resulting in antigenic diversity, which forms the basis of the widely used nucleotide-based *emm* typing scheme.^14^ Different *emm* types have distinct levels of pathogenicity or virulence, which may account for the diversity of infections in different settings.^13^

Penicillin remains the drug of choice for GAS infections; however, a vaccine will be the best option to control the burden of GAS diseases.^14^ An M-protein-based vaccine containing 30 different M-protein peptide molecules (*emm* types) is currently in the clinical trial stage of development.^15,16^ The vaccine coverage is dependent on the *emm types* circulating within a specific region, although there may be some cross-protection against non-vaccine *emm* types.^16^ A challenge in producing an effective type-specific M-protein-based vaccine is that there are more than 200 *emm*-types of GAS worldwide.^14^ The distribution and prevalence of these *emm* types vary across different geographic regions and may also change in a specific region over time.^17^

Other potential GAS vaccine candidates include superantigens (SAgs) that have been shown to play a vital role in the pathogenesis of infections.^5^ Superantigens are bacterial exotoxins with the ability to elicit an excessive T-cell response.^5^ Eleven streptococcal superantigen exotoxins (spe) have been reported in GAS namely, SpeA, SpeC, SpeG, SpeH, SpeI, SpeJ, SpeK, SpeL, SpeM, SS_A and streptococcal mitogenic exotoxin Z (SmeZ).^5^ The distribution of SAg genes varies by *emm* type and the region of the isolate’s origin.^18,19^ Not much is known about the SAg profiles of GAS in South Africa. Therefore, this study aimed to describe the prevalence of GAS pharyngitis in the northwestern region of Tshwane and to characterise GAS isolates by determining the antimicrobial susceptibility profiles, the circulating *emm* types and the SAgs associated with the *emm* types.

## Research methods and design

### Specimen and isolate collection

This was a cross-sectional descriptive quantitative study to investigate the prevalence of GAS pharyngitis in northwestern Tshwane and characterise GAS isolates from this region. Throat swabs were collected from children and young adults aged 1–22 years presenting with pharyngitis at a primary healthcare clinic in northwestern Tshwane. In addition, GAS clinical isolates from patients aged 1–66 years cultured at the Dr. George Mukhari Academic/National Health Laboratory Service (DGM laboratory), were collected during years 2019–2022. Patient information, including demographic data, was obtained from the Laboratory Information System–TrakCare (LIS).

### Bacterial culture

Throat swabs were cultured on 5% sheep blood agar (Diagnostic Media Products, South Africa) and incubated at 35 ± 2°C in 5% CO_2_ for 18–24 h. The resulting colonies were screened by standard microbiological tests, including sensitivity to bacitracin, to determine the presence of GAS. The Streptex agglutination test (Thermo Fisher Scientific, United Kingdom) was used to identify GAS by detecting the presence of the group A antigen of the organism. Pure GAS colonies were stored in Microbanks® (Pro-Lab Diagnostics, Canada) at –20°C for further characterisation. Clinical isolates were confirmed using the same standard microbiological tests used to identify GAS from the throat swabs.

### Antimicrobial susceptibility testing

Antibiotic susceptibility testing was performed using the E-test method to determine the minimum inhibitory concentrations (MICs). Briefly, a standardised inoculum (0.5 McFarland) of each GAS isolate was streaked on Mueller Hinton agar supplemented with 5% sheep blood (DMP, South Africa). Antibiotic test strips: penicillin, erythromycin and clindamycin (Liofilchem srl, Italy) were applied to the agar surface and incubated overnight at 35°C in 5% CO_2_. After incubation, the MICs were read and interpreted according to the Clinical & Laboratory Standards Institute (CLSI) guidelines.^20^

### *Emm* gene typing and exotoxin gene profiling

To determine the *emm* types, polymerase chain reaction (PCR) amplification and sequencing of the *emm* gene were performed. The genomic DNA of the GAS isolates was extracted using the boiling method, as previously described by Dashti et al.^21^ Conventional PCR was used for amplification of the *emm* gene using primers and conditions previously described.^22^ All primers used in this study were synthesised by Inqaba Biotechnical Industries (Pty), Ltd., Pretoria, South Africa. The PCR products were separated using 1% agarose gel electrophoresis run at 110 volts for 60 min. The gel images were visualised and captured using a Gel Doc^TM^ EZ system (Bio-Rad, United States). The GAS strain ATCC® 19615™ was used as a positive control, and *Streptococcus pneumoniae* (ATCC® 49619™) was used as a negative control.

Nucleic acid sequencing was performed at Inqaba Biotechnologies (Pty), Ltd., and the sequences generated were analysed using BioEdit version 7.1.1 (Bioscience, United States). The sequences were then subjected to a homology search on the Centers for Disease Control and Prevention (CDC) and the National Center for Biotechnology Information (NCBI) basic local alignment search tools. To predict potential vaccine coverage, the different *emm* types were then compared with the types included in the 30-valent vaccine.

Superantigen profiles were determined using conventional PCR amplification of 11 SAg genes (*speA, speB, speC, speG, speH, speI, speJ, speK, speL, speM, ssa, smeZ*) using previously published primers and the amplification conditions.^18,23^

### Data analysis

Descriptive statistical analysis was used to measure the frequencies of categorical data (e.g. antimicrobial susceptibility profiles, the occurrence of *emm* types and SAgs) and central tendency and dissemination of continuous data (e.g. age). A *p*-value of < 0.05 was considered significant. All the statistical analyses were performed using STATA® v18 (StataCorp, TX, United States).

### Ethical considerations

The study was approved on 03 October 2019 by the Sefako Makgatho Health Sciences University Research and Ethics Committee (reference number SMUREC/M/298/2019: PG) and the Gauteng provincial ethics committee. Permission to have access to the laboratory database and the isolates to be used for the study was obtained from the management of the DGM laboratory. Consent and assent were obtained from patients, parents and patient carers where necessary.

## Results

A total of 400 throat swabs were collected from patients who presented with symptoms of pharyngitis. Among these throat swabs, GAS was isolated from 33, resulting in a prevalence rate of 8%. The mean age of the patients was 11 years, with a greater proportion of females (21/33, 58%) compared to males (12/33, 42%) (*p* < 0.05). Additionally, 72 clinical GAS isolates were collected from the DGM laboratory. The patients’ ages ranged from 9 months to 66 years, with a mean age of 32 years. The gender distribution revealed that most of the patients were female, accounting for 63% (41/65), whereas 37% (24/65) were male (*p* < 0.05). The gender for 10% (7/72) of the patients was not known.

Overall, 105 GAS isolates were available for analysis; among these, 7.6% (8/105) were invasive, and 92.4% (97/105) were from non-invasive specimens. Most of the non-invasive isolates were recovered from the following specimens: superficial skin swabs (47), throat swabs (33) and pus (13). Notably, most of the invasive isolates were recovered from blood (3) and tissue (3) specimens, with the least from the bronchial aspirate (1), and wound (1). None of the isolates were resistant to penicillin, erythromycin and clindamycin, as shown in Table 1.

**TABLE 1:** Susceptibility of group A *Streptococcus* isolates to antimicrobial drugs using E-test method (*N* = 105).

Penicillin			Clindamycin			Erythromycin		
MIC range (µg/mL)	No. of isolates	Interpretation	MIC range (µg/mL)	No. of isolates	Interpretation	MIC range (µg/mL)	No. of isolates	Interpretation
0.004	6	S	0.016	1	S	0.016	1	S
0.008	29	S	0.032	2	S	0.032	10	S
0.016	56	S	0.064	21	S	0.064	65	S
0.032	6	S	0.12	40	S	0.12	18	S
0.064	5	S	0.25	37	S	0.25	9	S
0.12	3	S	0.5	4	I	0.5	2	I

Note: Interpretation of MIC range (µg/mL)^20^ for penicillin: S (≤ 0.12), R (> 0.12); clindamycin: S (≤ 0.25), I (0.5), R (> 1); erythromycin: S (≤ 0.25), I (0.5), R (> 1). Please see full reference list of this article, https://doi.org/10.4102/sajid.v40i1.714 for more information.

No., number; GAS, group A *Streptococcus*; MIC, minimum inhibitory concentration; S, Susceptible; R, Resistance; I, Intermediate.

### *Emm*-type distribution among group A *Streptococcus* isolates

A total of 27 different *emm* types were identified (Table 2). The most prevalent *emm* type was *emm82* (14%), followed by *emm68* (9%). Among the 27 *emm* types, 12 of the *emm* types are included in the 30-valent vaccine. The vaccine coverage was 48.6%, accounting for the isolates that are directly covered by the 30-valent vaccine; while the hypothetical vaccine coverage was 79% accounting for the *emm* types directly covered in the vaccine as well as the potential cross-opsonised non-vaccine types.

**TABLE 2:** *Emm* type distribution and 30-valent vaccine coverage among 105 invasive and non-invasive group A *Streptococcus* isolates collected in northern Tshwane, South Africa, 2019–2022.

*Emm* type	Isolates		Specimen type	Vaccine coverage
	*n*	%		
82	16	15.2	Pus swab (2), superficial skin swab (11), blood (1), throat swab (2)	VT
68	9	8.6	Superficial skin swab (2), wound swab (1), pus swab (6)	NVT†
22	7	6.7	Superficial skin swab (2), throat swab (5)	VT
1	6	5.7	Superficial skin swab (3), throat swab (3)	VT
25	6	5.7	Superficial skin swab (6)	NVT
66	6	5.7	Superficial skin swab (5), tissue (1)	NVT†
9	5	4.8	Superficial skin swab (2), throat swab (2), pus swab (1)	NVT†
63	5	4.8	Superficial skin swab (3), throat swab (1), tissue (1)	NVT
75	5	4.8	Pus swab (1), sputum (1), throat swab (3)	VT
49	4	3.8	Superficial skin swab (3), blood (1)	VT
65	4	3.8	Superficial skin swab (4)	NVT†
94	4	3.8	Throat swab (4)	NVT†
12	3	2.9	Throat swab (3)	VT
57	3	2.9	Superficial skin swab (1), throat swab (2)	NVT
58	3	2.9	Superficial skin swab (1), throat swab (1), pus swab (1)	VT
80	3	2.9	Superficial skin swab (3)	NVT
87	3	2.9	Throat swab (3)	VT
90	3	2.9	Superficial skin swab (3)	NVT
33	2	1.9	Superficial skin swab (1), throat swab (1)	NVT†
3	1	0.9	Throat swab (1)	VT
4	1	0.9	Throat swab (1)	VT
28	1	0.9	Superficial skin swab (1)	VT
44	1	0.9	Throat swab (1)	VT
48	1	0.9	Tissue (1)	NVT†
55	1	0.9	Blood (1)	NVT
79	1	0.9	Bronchial aspirate (1)	NVT†
119	1	0.9	Sputum (1)	NVT

Note: Please see full reference list of this article, https://doi.org/10.4102/sajid.v40i1.714 for more information.

VT, Vaccine type; NVT, Nonvaccine type.

† , Cross-opsonised (> 40% bactericidal killing) in animal studies.^16^

### Superantigens in the group A *Streptococcus* isolates

Ten SAgs were detected among the 105 GAS isolates (Table 3). The most common SAgs were *speG* (82.9%, 87/105) and *smeZ* (81.6%, 86/105). *SpeK* was found at the lowest frequency (6.7%; 7/105), whereas *speL* and *speM* were not detected in any of the isolates. *speH* and *speI* were more prevalent in *emm*82 isolates, each detected in 81.25% of cases (13/16), indicating equal prevalence (Table 3). Five GAS isolates did not have any SAg profile detected. The presence of SAgs was also correlated with the *emm* types.

**TABLE 3:** The distribution of superantigens among *emm* types of invasive and non-invasive group A *Streptococcus* isolates collected in northern Tshwane, South Africa, 2019–2022 (*N* = 105).

Superantigens	Isolates		*Emm* types (number of isolates)
	*n*	%	
*speG*	87	82.9	82(10), 1(6), 68(6), 22(5), 25(5), 66(5), 75(5), 9(4), 63(4), 12(3), 49(3), 57(3), 58(3), 65(3), 80(3), 87(3), 90(3), 94(3), 33(2), 3(1), 4(1), 28(1), 44(1), 48(1), 55(1), 79(1), 119(1)
*smeZ*	86	81.9	82(12), 1(6), 22(5), 66(5), 75(5), 9(4), 25(4), 63(4), 68(4), 12(3), 49(3), 57(3), 58(3), 65(3), 80(3), 87(3), 90(3), 94(3), 33(2), 4(1), 3(1), 28(1),44(1), 48(1), 55(1), 79(1), 119(1)
*speH*	41	39.1	82(13), 63(5), 49(4), 94(4), 12(3), 25(3), 33(2), 44(1), 55(1), 57(1), 58(1), 65(1), 119(1)
*speI*	34	32.4	82(13), 49(4), 12(3), 25(2), 66(2), 75(2), 1(1), 22(1), 33(1), 44(1), 55(1), 58(1), 65(1), 79(1), 94(1)
*speC*	25	23.8	66(5), 22(4), 75(4), 90(3) 12(2), 57(2), 82(2), 1(1), 58(1)
*speA*	21	20.0	1(5), 9(2), 22(2), 33(2), 80(2), 82(2), 4(1), 12(1)) 44(1), 49(1), 55(1), 87(1)
*speJ*	14	13.3	1(4), 80(3), 25(3), 9(2), 28(1), 44(1), 82(1)
*Ssa*	8	7.6	9(2), 33(2), 4(1), 22(1), 57(1), 87(1)
*speK*	7	6.7	75(5), 80(2)
*speL and speM*	0	-	-

The SAgs were further grouped into distinct genotypic profiles according to previously reported nomenclature.^18,19,23^ Among the 105 isolates, 26 SAg profiles were found, of which the most common profiles were K and Q with 10 isolates each (Table 4). Three *emm* types (*emm*12, *emm*22 and *emm*75) were covered in three or more different SAg profiles (Table 4).

**TABLE 4:** *Emm* type distribution among 105 invasive and non-invasive group A *Streptococcus* isolates collected in northern Tshwane, South Africa, 2019–2022.

Profile	*Emm* type	*speG*	*smeZ*	*speH*	*speI*	*speC*	*speA*	*SpeJ*	*Ssa*	*speK*	No. of isolates
Q	82	+	+	+	+	-	-	-	-	-	8
	65	+	+	+	+	-	-	-	-	-	1
	25	+	+	+	+	-	-	-	-	-	1
K	63	+	+	+	-	-	-	-	-	-	5
	94	+	+	+	-	-	-	-	-	-	3
	25	+	+	+	-	-	-	-	-	-	1
	119	+	+	+	-	-	-	-	-	-	1
N	90	+	+	-	-	+	-	-	-	-	3
	66	+	+	-	-	+	-	-	-	-	3
	22	+	+	-	-	+	-	-	+	-	1
X	75	+	+	-	-	+	-	-	-	+	3
W	1	+	+	-	-	-	+	+	-	-	2
	9	+	+	-	-	-	+	+	-	-	2
	80	+	+	-	-	-	+	+	-	-	1
O	25	+	+	-	-	-	-	+	-	-	2
	28	+	+	-	-	-	-	+	-	-	1
M	66	+	-	-	+	+	-	-	-	-	2
B	22	+	+	-	-	-	-	-	-	-	1
	58	+	+	-	-	-	-	-	-	-	1
	68	+	+	-	-	-	-	-	-	-	1
I	49	+	-	+	+	-	-	-	-	-	1
	82	+		+	+	-	-	-	-	-	1
AI	12	+	+	+	+	-	+	-	-	-	1
	55	+	+	+	+	-	+	-	-	-	1
	82	+	+	+	+	-	+	-	-	-	1
L	79	+	+	-	+	-	-	-	-	-	1
	82	+	+	-	+	-	-	-	-	-	1
AA	9	+	+	-	-	-	+	-	+	-	1
	87	+	+	-	-	-	+	-	+	-	1
AM	12	+	+	+	+	-	-	-	-	-	1
	82	+	+	+	+	-	-	-	-	-	1
A	48	+	-	-	-	-	-	-	-	-	1
C	22	+	-	-	-	+	-	-	-	-	1
D	49	-	-	+	+	-	-	-	-	-	1
E	25	+	-	-	+	-	-	-	-	-	1
F	80	-	-	-	-	-	-	+	-	+	1
G	1	+	+	-	-	-	+	-	-	-	1
H	44	+	-	+	+	-	+	-	-	-	1
J	82	+	+	-	+	-	-	-	-	-	1
P	9	+	+	-	-	-	-	-	+	-	1
R	57	+	+	+	-	+	-	-	-	-	1
T	82	+	-	+	-	+	+	-	-	-	1
U	1	+	+	-	+	-	+	-	-	-	1
V	49	+	-	+	+	-	+	-	-	-	1
Y	25	+	+	+	-	-	-	+	-	-	1
Z	75	+	+	-	+	-	-	+	-	+	1
AB	12	+	-	+	+	+	-	-	-	-	1
AC	57	+	+	-	-	+	-	-	+	-	1
AD	4	+	+	-	-	-	+	-	+	-	1
AE	80	+	+	-	-	-	+	+	-	+	1
AF	22	+	+	-	-	+	+	-	+	-	1
AG	1	+	+	-	-	+	+	+	-	-	1
AH	33	+	-	+	-	-	+	-	+	-	1
AJ	22	+	+	-	+	+	+	-	-	-	1
AK	33	+	+	-	-	-	+	-	+	-	1
AL	75	+	+	-	+	+	-	-	-	+	1

+, Represents the presence of the superantigen; -, Represents the absence of the superantigen.

## Discussion

This study sought to describe the prevalence of GAS pharyngitis in the northwestern region of Tshwane and to characterise GAS isolates from this region. The study reported on antibiotic susceptibility profiles, circulating *emm* types and the SAg profiles of GAS isolates found in this region. The prevalence of pharyngeal GAS was 8%, which is lower than the 17% reported in another South African study performed in the same setting by Khosa et al.^11^ Another South African study in Cape town reported a higher prevalence rate of 21.6%.^24^ Studies from other African countries reported differing GAS prevalence rates. An Ethiopian study reported a prevalence of 9.1% among children younger than 18 years presenting with pharyngitis,^25^ whereas much higher prevalence rates of 42.2% and 32.9% were reported in Egypt and Tunisia, respectively.^26,27^ These reports indicate that there is great variability in the prevalence of GAS in different regions at different times.^28^

Despite the development of penicillin resistance among other pathogens such as *Streptococcus pneumoniae*, GAS is one of the few pathogens that remain susceptible to the antibiotic, which makes it the antibiotic of choice for treating GAS infections.^12,13,29,30^ All the isolates in the present study were susceptible to penicillin, with MICs ranging from 0.004 µg/mL to 0.094 µg/mL. Despite the fact that GAS remains susceptible to penicillin, it is important to continually monitor the possible gradual increase of MICs over time to detect any indication of early resistance. There was no resistance observed to clindamycin or erythromycin, which are used as alternatives in cases of penicillin allergy.^12^ However, 4 (3.8%) and 2 (1.6%) of the isolates presented MICs above the susceptible range for clindamycin and erythromycin, respectively. These findings support and align with reports indicating that GAS remains fully susceptible to penicillin, while MICs of clindamycin and erythromycin are on the rise.^25,31^ Notably, resistance to these agents has already been reported in other regions such as China, Finland, Greece, Spain and the US.^1,32^

This study identified 27 distinct *emm* types across various infection sites, including 15 *emm* types isolated from patients with pharyngitis. In another study conducted in the same setting in 2021, a total of 15 different *emm* types from 54 invasive and non-invasive isolates recovered from multiple sites of infections were reported.^11^ However, the *emm* type distribution differed significantly across studies conducted in the same region over different years. Another study performed in Cape Town (South Africa) reported 35 different *emm* types among isolates from children aged 3–15 years who also presented with pharyngitis.^33^ In another region of the African continent, a study conducted in Ethiopia identified 43 distinct *emm* types in 82 GAS isolates colonising healthy children aged 6–14 years.^34^ A different picture of the circulating *emm* types is reported in the high-income continents of Europe and North America, with *emm1* reported to be the most prevalent in both continents.^35^ The 30-valent vaccine was specifically designed to contain *emm* types that are prevalent in the US, Canada and Europe, with a potential coverage of 85% of pharyngitis cases and invasive infections in these regions.^36^

In this study, the most prevalent *emm* type was *emm*82, and among the pharyngeal isolates, *emm*22 (15%) and *emm*94 (12%) were the most prevalent. These findings contrast those of two previous studies performed in different regions of South Africa by Khosa et al.^11^ and Engel et al.^24^ The study by Engel et al. in Cape Town reported that *emm*48 was the most prevalent *emm* type followed by *emm*22 and *emm*94, which made up most of the pharyngeal isolates.^24^ A study conducted by Khosa et al., in Tshwane, reported *emm92* as the most prevalent *emm* type of the total collected GAS isolates, regardless of the site of infection^11^ whereas in the present study, *emm*92 was not detected at all. Although there is a difference in the most prevalent *emm* types, there is an overlap in the overall *emm* types circulating in these regions, especially *emm* types (*emm*22 and *emm*94), which were detected in both studies. These findings support the notion that there is diversity in the *emm* types circulating in different regions, even within the same country.^17^

Among the 27 *emm* types identified in the present study, 12 (44.4%) were vaccine types, and 15 (55.6%) were nonvaccine types. Therefore, the vaccine coverage was 48.6% accounting for the isolates that are directly covered by the 30-valent vaccine, which may have implications in the development of a vaccine for this region with this low vaccine coverage. However, the hypothetical vaccine coverage was 79%, which includes both the *emm* types directly targeted by the vaccine and those potentially cross-opsonised. Among the 54 GAS isolates characterised by Khosa et al., 23% of the *emm* types were reported as vaccine types while 67% were non-vaccine types.^11^ However, no hypothetical vaccine coverage was determined to predict potential coverage, including the opsonised non-vaccine types. Engel et al. estimated that the 30-valent potential vaccine coverage was 95% of pharyngitis cases in the Cape Town population at risk of rheumatic fever, which is in contrast to our findings, where only 48.6% of the potential vaccine coverage is found among the isolates collected in this region.^16^

Superantigens play a vital role in the pathogenesis of diseases, and thus, knowledge of their profiles will enhance prevention and control strategies.^37^ In the present study, *speG* was detected in the majority (82.9%) of the isolates, followed by *smeZ* (81.90%). The *speG* and *smeZ* genes are reported to be located on the core chromosome, and hence, they are expected to be present in nearly all isolates.^37,38^ Some studies have reported 100% detection of *speG*,^33,38,39,40^ while a study conducted in Cape Town by Muhamed reported *speG* in 94% of their isolates.^33^ *SpeJ* (chromosomally encoded) and *speA* (putative bacteriophage encoded) are both reported to be associated with invasive infections.^4^ The present study reported a 13% prevalence of *speJ* among non-invasive isolates and a 20% prevalence of *speA*, with the majority (76%) detected among non-invasive isolates. Muhamed in Cape Town reported an overall *speJ* prevalence of 66% and an overall *speA* prevalence of 17%, whereas Ekelund in Denmark reported a 34% prevalence of *speA* in invasive isolates.^33,38^ However, they found no associations between any particular SAgs, consistent with the findings in this study.^33^ In contrast to the above studies, in the UK, *speA* was found in mostly invasive isolates (70%) but in a minority of non-invasive isolates (11%), while the *speJ* gene was more common in invasive isolates (80%) than in non-invasive isolates (15%).^4^ A study conducted in Germany by Imöhl et al. reported 50.9% *speJ* in isolates recovered from invasive specimens.^13^

Reports have also indicated that most *emm* strains are associated with multiple SAgs.^18,38,41^ This was also found in the present study, where three *emm* types (*emm12, emm22* and *emm75*) were associated with three or more different SAgs, namely, *speA, speG* and *smeZ*. However, a study conducted in Belgium reported only *emm1* to be associated with multiple SAgs (*speA, speB* and *speF*).^42^ In the study conducted in the UK by Alcolea-Medina, most *emm1* strains (78%) were associated with *speA* and *speI*, including all of the invasive *emm1* isolates (100%).^4^ In the present study, *ssa* was not found to be associated with *emm*1 isolates, supporting the findings of Vlaminckx et al., who reported not having found *ssa* and *speC* to be associated with *emm1*.^43^

The different SAgs may be grouped into genotypic evolutionary groups.^18,19,23^ In this study, 39 different SAg profiles were identified from the 105 GAS isolates, and SAg profiles K and Q were the dominant profiles. In a study conducted in Cape Town, 21 different SAg profiles were identified in 98 GAS isolates.^33^ The study reported that *emm1* was associated with three SAg profiles (profiles A, E and F) as classified by Maripuu et al.^23,33^ In contrast to the Cape Town study, the present study identified *speA, speG* and *smeZ* as part of genotype profile G.^33^ This profile, previously reported as R by Commons et al. and Berman et al., was found to be the most prevalent in all *emm1* isolates, including the invasive isolates.^18,19^ The present study has found that *emm22* possesses three or more different SAg profiles (Table 4), which are the only *emm* types with a diverse SAg profile. The study findings suggest that the SAg profiles and *emm* type associations are not conserved.^43^

### Limitations of the study

A small sample size was collected from a single public health clinic during the coronavirus disease 2019 (COVID-19) lockdown period (2020–2021), when limited patient visits were allowed. This restriction affected the sampling process, preventing any statistical inferences from being drawn. Additionally, inducible clindamycin resistance testing was not performed, which may have implications for treatment decisions.

### Recommendations

More data are needed from low-income countries, including South Africa, where GAS pharyngitis and ARF/RHD are common, to inform vaccine composition. Alternative vaccine targets such as SAgs should be further explored. The antimicrobial susceptibility profiles of GAS should be monitored for the emergence of reduced susceptibility to penicillin.

## Conclusion

This study reported a GAS prevalence rate of 8% in patients with pharyngitis. The results of this study indicate that penicillin remains the drug of choice for the treatment of GAS infections. Considerable diversity of *emm* types was observed in this area, with *emm*82 being the most prevalent. Hypothetically, the potential 30-valent vaccine would cover 79% of the isolates found in this region. Superantigen profiling revealed a diverse profile of SAg genes, with K and Q being the most prevalent. Superantigens might not be viable vaccine targets because of their diversity; therefore, further exploration can be conducted to produce a viable SAg vaccine that will reduce the burden of GAS infections.
